# Round the Hospitals

**Published:** 1916-07-08

**Authors:** 


### ROUND THE HOSPITALS.

The Queen looked her very best, and Her Majesty was a charming, tactful personality on entering Queen Mary Ward on her way to the platform. She seemed conscious that the South London Hospital for Women, with its eighty beds, is wholly a women's hospital, that it is managed and staffed by women, and that Her Majesty's wish was to identify herself specially with this institution on that account. It was very gracious of the Queen to go cheerfully through six different ceremonies in the Lower Hall, the Upper Hall, the Queen Mary Ward, the pavilion downstairs, the unveiling of the stone in the courtyard, and the inspection of the buildings. The restricted site, and the consequent planning of the buildings, did not facilitate these numerous ceremonies, and we hope the authorities were duly appreciative of the unusual honour they had conferred upon them by Her Majesty in undertaking six ceremonies in one day in support of their hospital. We have always found it to be a mistake to open new hospitals, and to exhibit new wards equipped with makeshift furniture and fittings, when those selected for the new hospital are not ready, as was the case at Clapham Common on the 4th instant. The operation unit, as will be seen on studying the plan and description on page 349, is not satisfactory. Before the hospital is opened we hope it may be rearranged, so as to remove some patent defects which it exhibited on the opening day. There are other matters which call for adjustment too, as we have no doubt the matron and staff will demonstrate and have put right before the serious work is entered upon. For the rest, we congratulate those responsible for the arrangements, and commend especially the idea of inviting those present to wear their college gowns and hoods, for they gave a bright and effective air to the proceedings. We also congratulate the Marchioness of Londonderry on the brevity of her address to the Queen, which might with advantage be taken as a model for future use by other institutions. Quite a large number of purses were handed to Her Majesty, nearly thirty of which had been raised by societies, schools, patients' and teachers' leagues, and other leagues of more than one political party. The matron, Miss Jones Pearse, was indefatigable in her efforts to make everyone at their ease. Dr. Margaret Fraser, the senior physician, Miss Chadburn, B.S., senior surgeon, Miss Ridler, the secretary, and the Matron were presented to the Queen by the Marchioness of Londonderry, who is chairman of the hospital.

---

The so-called conference of members of Nurses' Societies and Leagues, summoned by the National Union of Trained Nurses on June 29, if judged by the attendance, must have been disappointing to its promoters. It was announced that four questions of the day, as they were called, would be raised in ten-minute papers by selected authorities. Most of the papers were not forthcoming, but Mrs. Strong presented one on the difficulties of training in small institutions, and Miss Cancellor another on the possibilities of combined training. One point she made was that cottage hospitals took probationers from the larger hospitals. In practice, the probationers at the former are young girls and so disqualified by age to join the larger institutions. Miss Cancellor is apparently unaware that the best cottage hospitals have an arrangement with county hospitals which constitutes the former a recruiting ground for the latter through the co-operation of the matrons concerned. In fact, one of the most efficient, up-to-date, and remarkable cottage hospitals in the country, owing to the value of its clinical and surgical work, has had such an arrangement for years, which has worked admirably.

---

The plan referred to does not in any way interfere with the real training of the woman who has spent a year in the cottage hospital before attaining an age to qualify for the larger institution. This system obviates the objections properly insisted upon by Miss Musson (Birmingham General Hospital) and Miss Gibson (Poor Law), as the cottage hospital period in no way shortens the full three years' training at the general hospital, which these women enter as ordinary probationers. The safeguard and help for the general hospital matron which this system provides is her knowledge of the cottage hospital matron, whom she can trust to recommend her suitable probationers only. This is the key to the difficulties mentioned, and has the merit of keeping the younger women interested in hospital work till they are of an age to enter upon a full course of training. The details of this plan can safely be left to the matrons of the larger hospitals, who have trusted members of their training school acting as matrons in cottage hospitals. The cottage hospital matrons who have successfully adopted this system practically perform the part of the sister in a large hospital *vis-à-vis* with its matron. Such a sister would not take an older nurse as a probationer because her experience has taught her that they do not succeed in cottage hospitals, and because a certain number of this class of institution have been giving two years' certificates of training without possessing the means to train at all in any proper meaning of the word. We hold that the College of Nursing, if it is to succeed, must put a stop to the issue of certificates by the authorities of any institution which has not the facilities or the means completely to train nurses for the sick. Mrs. Strong's properly urged difficulties would also be removed.

---

At the conference of members of Nurses' Societies held on the 29th ultimo, in dealing with the political position in the nursing world, it is interesting to record that Mrs. Bedford Fenwick claimed that she had been the means of bringing about the conferences between the College of Nursing and the State Registrationists, which promise to result in an agreed Bill and so to close a controversy which has split the nursing profession and those interested in it for decades of years. This was a large claim, and how far it will be found to be justified the history of the College of Nursing and of the Hon. Arthur Stanley's work and that of those associated with him in the enterprise, when published, will no doubt demonstrate.

For Matrons' and Sisters' Appointments see page vi. For College of Nursing Official Notices see page 358.

